# Supplementary material for: Extended Versus Intermittent Meropenem Infusion in the Treatment of Nosocomial Pneumonia: A Retrospective Single-Center Study
Source: Antibiotics (Basel). 2023 Oct 15;12(10):1542. doi: 10.3390/antibiotics12101542 (PMC10604670; doi:10.3390/antibiotics12101542)
Supplement: Supplementary file 1 [file antibiotics-12-01542-s001.zip › antibiotics-2575080-supplementary.pdf]

antibiotics

Supplementary Materials

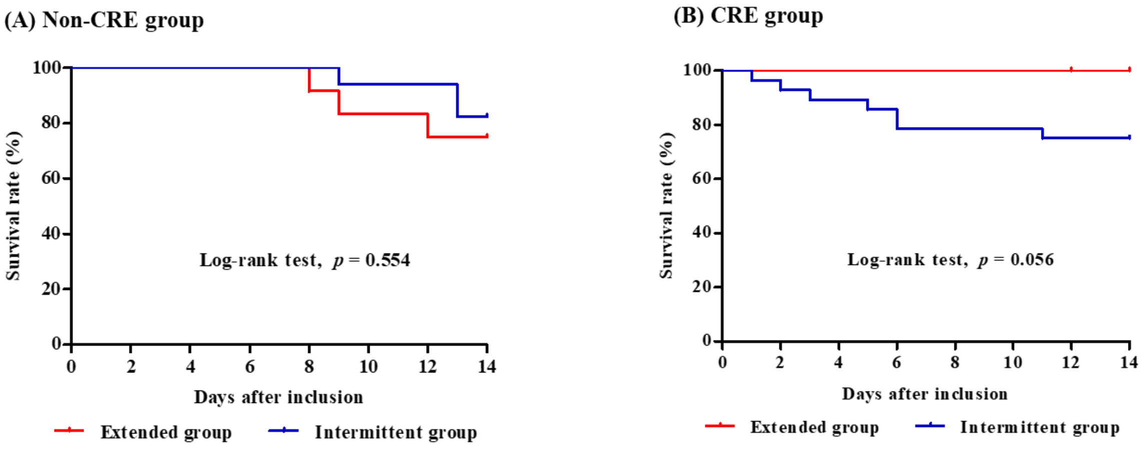

Figure S1. Mortality in subgroups at 14 days. (**A**) Non-carbapenem-resistant *Enterobacteriaceae* (CRE) and (**B**) CRE. CRE, carbapenem-resistant *Enterobacteriaceae.*

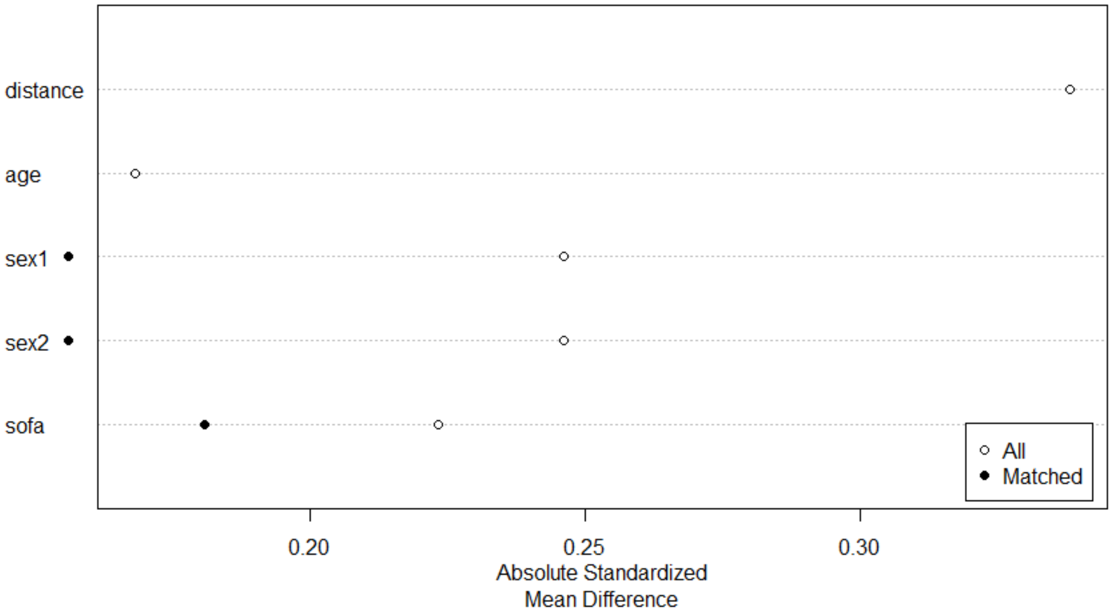

Figure S2. Absolute standardized mean differences between the extended infusion group and intermittent infusion group before and after propensity-score matching. Sofa; Sequential organ failure assessment.

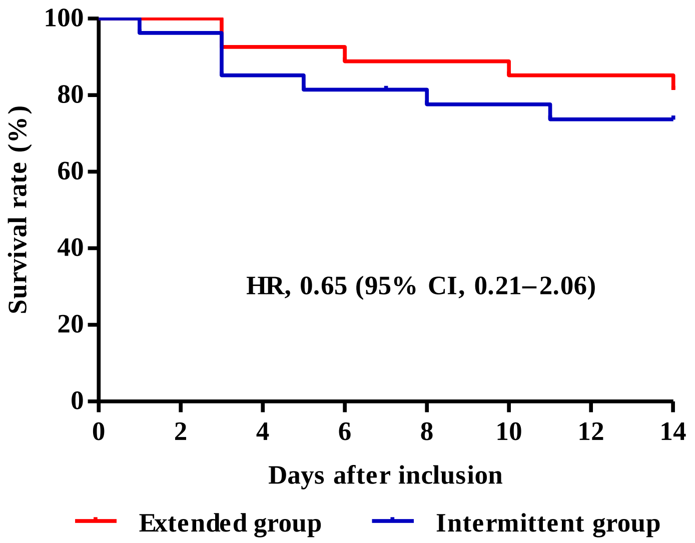

Figure S3. Time-to-event analysis after inclusion to day 14 in the propensity-score matched cohort. HR, hazard ratio; CI, confidence interval.

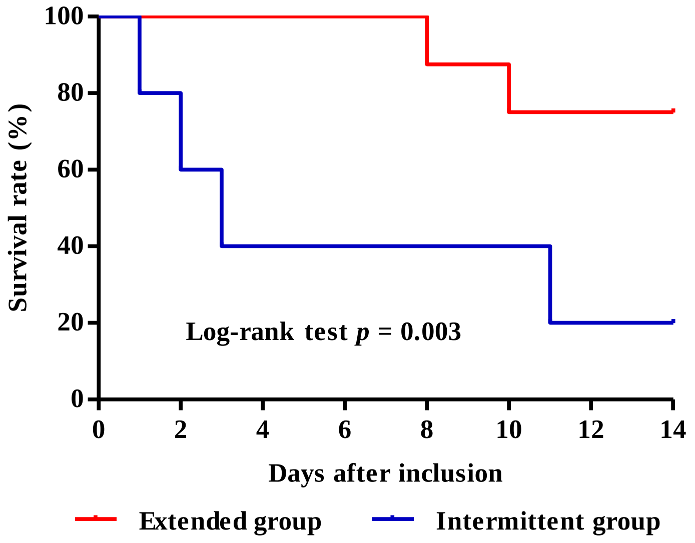

Figure S4. Time-to-event analysis after inclusion to day 14 in patients with concurrent bacteremia.

Table S1. Cox proportional hazards model of factors associated with mortality in all patients at 14 days.

Variable	Univariable Analysis			Multivariable Analysis		
	HR	95% CI	*p*-value	HR	95% CI	*p*-value
**Extended infusion**	**0.65**	**0.31–1.37**	**0.260**	0.55	0.23–1.31	0.174
Age, y	1.00	0.97–1.02	0.717			
Sex, male	1.66	0.81–3.39	0.166			
BMI, kg/m^2	1.01	0.94–1.09	0.753			
Co-existing condition						
Diabetes mellitus	0.60	0.26–1.39	0.232			
**Solid cancer**	**3.22**	**1.61–6.45**	**0.001**	2.22	1.08–4.56	0.029
Hematologic malignancy	0.53	0.19–1.51	0.236			
Congestive heart failure	0.34	0.05–2.51	0.291			
Respiratory failure	0.45	0.16–1.29	0.137			
**Chronic liver disease**	**2.07**	**0.89–4.79**	**0.089**	0.96	0.33–2.79	0.936
End-stage renal disease	0.41	0.10–1.73	0.227			
Immunocompromised	0.54	0.22–1.31	0.173			
Severity of pneumonia						
**SOFA score**	**1.27**	**1.13–1.42**	**<0.001**	1.19	1.02–1.39	0.024
Sepsis	1.80	0.25–13.20	0.562			
**Septic shock**	**2.86**	**1.43–5.74**	**0.003**	1.59	0.68–3.72	0.288
PaO2/FiO2 ratio, mmHg	1.00	0.99–1.00	0.167			
Type of pneumonia						
**VAP (vs. HAP)**	**2.33**	**1.15–4.72**	**0.019**	2.83	1.28–6.26	0.010
Parameters of antibiotics						
Previous antibiotic						
Class of penicillin	1.35	0.68–2.71	0.391			
Class of cephalosporin	1.28	0.55–2.96	0.564			
**Class of quinolone**	**2.26**	**1.13–4.53**	**0.021**	1.78	0.85–3.71	0.125
**Class of glycopeptide**	**2.57**	**1.26–5.26**	**0.010**	1.83	0.84–3.99	0.128
Concomitant antibiotic	3.66	0.50–2.69	0.201			
Use of colistin	0.67	0.20–2.18	0.502			
Coverage for MRSA	1.90	0.82–4.40	0.133			
Laboratory variables						
White cell count, × 10^3/L	1.01	0.97–1.05	0.554			
C-reactive protein, mg/dL	0.99	0.95–1.03	0.620			
Procalcitonin, ng/mL	0.99	0.94–1.04	0.674			
Lactate level, mmol/L	1.10	0.99–1.21	0.065			
Organ support at baseline						
Mechanical ventilation	23.38	0.13–4252.45	0.235			
RRT	1.67	0.79–3.53	0.177			
ECMO	0.98	0.34–2.78	0.964			
Vasopressor	2.06	0.93–4.59	0.076			
Microbiologic profile						
**Bacteremia**	**3.44**	**1.41–8.36**	**0.006**	7.14	2.29–22.31	0.001
Pathogen of sputum culture						
Gram(+) pathogen	0.45	0.06–3.31	0.435			
Gram(-) pathogen	0.89	0.44–1.81	0.756			

Abbreviations: HR, hazard ratio; CI, confidence interval; BMI, body mass index; SOFA, sequential organ failure assessment; PaO2, partial oxygen pressure in arterial blood; FiO2, fraction of inspired oxygen; VAP, ventilator-associated pneumonia; HAP, hospital-acquired pneumonia; MRSA, methicillin-resistant *Staphylococcus aureus*; RRT, renal replacement therapy; ECMO, extracorporeal membrane oxygenation.

Table S2. Cox proportional hazards model of factors associated with ICU discharge in all patients at 14 days.

Variable	Univariable Analysis			Multivariable Analysis		
	HR	95% CI	*p*-value	HR	95% CI	*p*-value
**Extended infusion**	**0.913**	**0.56–1.49**	**0.72**	0.767	0.4366–1.348	0.36
Age, y	0.996	0.98–1.01	0.66			
Sex, female	1.04	0.63–1.74	0.87			
BMI, kg/m^2	1.03	0.97–1.09	0.37			
Co-existing condition						
Diabetes mellitus	0.855	0.52–1.4	0.53			
**Solid cancer**	**1.61**	**0.95–2.73**	**0.08**	1.777	0.8385–3.764	0.1300
**Hematologic malignancy**	**1.94**	**1.18–3.18**	**0.009**	1.866	0.9982–3.489	0.0510
Congestive heart failure	0.514	0.18–1.50	0.22			
**Respiratory failure**	**0.459**	**0.24–0.86**	**0.022**	0.809	0.3603–1.816	0.6100
End-stage renal disease	0.782	0.36–1.71	0.54			
Chronic liver disease	1.04	0.50–2.17	0.92			
Immunocompromised	1.19	0.72–1.96	0.50			
Severity of pneumonia						
SOFA score	0.992	0.93–1.06	0.82			
Sepsis	0.472	0.19–1.18	0.110			
Septic shock	1.38	0.76–2.47	0.272			
PaO2/FiO2 ratio, mmHg	1.00	0.99–1.000	0.25			
Type of pneumonia						
VAP (vs. HAP)	0.772	0.47–1.28	0.31			
Parameters of antibiotics						
Previous antibiotic						
Class of penicillin	1.06	0.66–1.71	0.81			
Class of cephalosporin	1.0	0.55–1.84	0.99			
**Class of quinolone**	**1.65**	**1.01–2.70**	**0.045**	1.273	0.7520–2.155	0.3700
Class of glycopeptide	0.72	0.36–1.44	0.35			
Concomitant antibiotic						
**Use of colistin**	**0.215**	**0.05–0.86**	**0.03**	0.245	0.0515–1.171	0.0780
Coverage for MRSA	1.31	0.75–2.3	0.35			
Laboratory variables						
White cell count, × 10^3/L	1.01	0.98–1.04	0.39			
C-reactive protein, mg/dL	1.01	0.99–1.04	0.33			
Procalcitonin, ng/mL	0.985	0.96–1.01	0.19			
**Lactate level, mmol/L**	**1.04**	**1.00–1.09**	**0.049**	1.018	0.9653–1.073	0.5100
Organ support at baseline						
**Mechanical ventilation**	**0.385**	**0.20–0.75**	**0.005**	0.355	0.1738–0.724	0.0044
RRT	0.96	0.54–1.70	0.89			
ECMO	0.569	0.27–1.19	0.13			
Vasopressor	1.17	0.71–1.94	0.55			
Microbiologic profile						
Bacteremia	1.1	0.40–3.06	0.85			
Pathogen of sputum culture						
Gram(+) pathogen	0.435	0.11–1.68	0.23			
Gram(-) pathogen	0.661	0.397–1.1	0.11			

Abbreviations: HR, hazard ratio; CI, confidence interval; BMI, body mass index; SOFA, sequential organ failure assessment; PaO2, partial oxygen pressure in arterial blood; FiO2, fraction of inspired oxygen; VAP, ventilator-associated pneumonia; HAP, hospital-acquired pneumonia; MRSA, methicillin-resistant *Staphylococcus aureus*; RRT, renal replacement therapy; ECMO, extracorporeal membrane oxygenation.

Table S3. Cox proportional hazards model of factors associated with ventilator liberation in all patients at 14 days.

	Univariable Analysis			Multivariable Analysis		
Variable	HR	95% CI	*p*-value	HR	95% CI	*p*-value
**Extended infusion**	**1.07**	**0.62–1.82**	**0.82**	0.93	0.51–1.68	0.800
Age, y	0.99	0.97–1.01	0.34			
Sex, female	1.18	0.68–2.06	0.56			
BMI, kg/m^2	1.03	0.98–1.09	0.22			
Co-existing condition						
Diabetes mellitus	0.72	0.39–1.32	0.29			
Solid cancer	1.04	0.56–1.93	0.90			
Hematologic malignancy	1.33	0.72–2.45	0.36			
Congestive heart failure	0.72	0.29–1.77	0.47			
Respiratory failure	0.65	0.33–1.28	0.21			
End-stage renal disease	0.62	0.26–1.49	0.28			
Chronic liver disease	0.67	0.26–1.69	0.40			
Immunocompromised	1.11	0.63–1.96	0.72			
Severity of pneumonia						
SOFA score	0.956	0.90–1.02	0.18			
Sepsis	1.16	0.42–3.20	0.78			
Septic shock	1.69	0.94–3.04	0.08			
**PaO2/FiO2 ratio, mmHg**	**1.00**	**1.00–1.00**	**0.02**	1.00	0.99–0.99	1.00
Type of pneumonia						
VAP (vs. HAP)	1.05	0.61–1.81	0.86			
Parameters of antibiotics						
Previous antibiotic						
Class of penicillin	1.27	0.75–2.15	0.38			
Class of cephalosporin	0.74	0.34–1.64	0.46			
Class of quinolone	1.15	0.65–2.03	0.63			
Class of glycopeptide	0.69	0.35–1.37	0.28			
Concomitant antibiotic						
**Use of colistin**	**0.21**	**0.05–0.85**	**0.029**	0.24	0.06–1.01	0.052
Coverage for MRSA	1.47	0.82–2.64	0.20			
Laboratory variables						
White cell count, × 10^3/L	0.99	0.95–1.03	0.51			
C-reactive protein, mg/dL	1.01	0.98–1.04	0.60			
Procalcitonin, ng/mL	0.98	0.93–1.03	0.34			
**Lactate level, mmol/L**	**1.09**	**1.02–1.16**	**0.007**	1.02	0.93–1.13	0.670
Organ support at baseline						
Mechanical ventilation	0.77	0.38–1.55	0.46			
RRT	0.79	0.41–1.51	0.48			
ECMO	0.82	0.36–1.87	0.63			
Vasopressor	0.93	0.54–1.59	0.79			
Microbiologic profile						
Bacteremia	0.58	0.18–1.90	0.37			
Pathogen of sputum culture						
Gram(+) pathogen	0.48	0.12–1.99	0.31			
Gram(-) pathogen	0.80	0.47–1.36	0.41			

Abbreviations: HR, hazard ratio; CI, confidence interval; BMI, body mass index; SOFA, sequential organ failure assessment; PaO2, partial oxygen pressure in arterial blood; FiO2, fraction of inspired oxygen; VAP, ventilator-associated pneumonia; HAP, hospital-acquired pneumonia; MRSA, methicillin-resistant *Staphylococcus aureus*; RRT, renal replacement therapy; ECMO, extracorporeal membrane oxygenation.

Table S4. Cox proportional hazards model of factors associated with mortality at 14 days in the subgroup with empirical treatment.

Variable	Univariable analysis			Multivariable analysis		
	HR	95% CI	*p*-value	HR	95% CI	*p*-value
**Extended infusion**	**0.26**	**0.06–1.22**	**0.087**	0.17	0.03–0.96	0.045
Age, y	0.99	0.96–1.03	0.572			
Sex, female	1.50	0.40–5.64	0.552			
BMI, kg/m^2	0.98	0.84–1.16	0.838			
Co-existing condition						
Diabetes mellitus	1.35	0.40–4.62	0.630			
**Solid cancer**	**3.85**	**1.17–12.65**	**0.026**	2.11	0.52–8.55	0.297
Hematologic malignancy	1.28	0.34–4.81	0.720			
Congestive heart failure	0.04	0.00–419.35	0.502			
Respiratory failure	0.41	0.05–3.22	0.398			
End-stage renal disease	0.57	0.07–4.48	0.596			
Chronic liver disease	1.68	0.36–7.72	0.514			
Immunocompromised	0.76	0.20–2.85	0.680			
Severity of pneumonia						
**SOFA score**	**1.16**	**0.98–1.36**	**0.086**	1.19	0.96–1.48	0.114
Sepsis	21.82	0.00–	0.606			
Septic shock	2.23	0.65–7.63	0.202			
PaO2/FiO2 ratio, mmHg	0.99	0.99–1.00	0.673			
Type of pneumonia						
**VAP (vs. HAP)**	**7.68**	**2.33–25.34**	**0.001**	24.47	4.58–130.83	<0.001
Parameters of antibiotics						
Previous antibiotic						
**Class of penicillin**	**3.94**	**1.15–13.49**	**0.029**	0.19	0.03–1.35	0.097
Class of cephalosporin	0.88	0.11–6.87	0.902			
**Class of quinolone**	**4.86**	**1.28–18.44**	**0.020**	12.42	1.50–102.84	0.019
Class of glycopeptide	5.73	1.74–18.86	0.004			
Concomitant antibiotic						
Use of colistin	1.64	0.35–7.58	0.529			
Coverage for MRSA	1.98	0.43–9.14	0.384			
Laboratory variables						
White cell count, × 10^3/L	1.04	0.97–1.11	0.296			
C-reactive protein, mg/dL	1.03	0.96–1.10	0.444			
Procalcitonin, ng/mL	0.99	0.94–1.044	0.781			
Lactate level, mmol/L	1.06	0.92–1.23	0.404			
Organ support at baseline						
Mechanical ventilation	23.12	0.00–	0.502			
RRT	2.51	0.77–8.25	0.128			
ECMO	1.46	0.32–6.75	0.630			
Vasopressor	1.20	0.35–4.12	0.767			
Microbiologic profile						
Bacteremia	1.96	0.42–9.07	0.390			
Pathogen of sputum culture						
Gram(+) pathogen	0.05	0.00–5594.73	0.606			
Gram(-) pathogen	1.25	0.38–4.08	0.718			

Abbreviations: HR, hazard ratio; CI, confidence interval; BMI, body mass index; SOFA, sequential organ failure assessment; PaO2, partial oxygen pressure in arterial blood; FiO2, fraction of inspired oxygen; VAP, ventilator-associated pneumonia; HAP, hospital-acquired pneumonia; MRSA, methicillin-resistant *Staphylococcus aureus*; RRT, renal replacement therapy; ECMO, extracorporeal membrane oxygenation.

Table S5. Cox proportional hazards model of factors associated with mortality at 14 days in the subgroup of culture-proven patients.

	Univariable Analysis			Multivariable Analysis		
Variable	HR	95% CI	*p*-value	HR	95% CI	*p*-value
**Extended infusion**	**0.51**	**0.14–1.84**	**0.300**	0.10	0.01–0.83	0.033
Age, y	1.00	0.97–1.04	0.849			
Sex, female	1.24	0.41–3.81	0.701			
BMI, kg/m^2	1.02	0.89–1.16	0.794			
Co-existing condition						
Diabetes mellitus	0.53	0.12–2.38	0.406			
Solid cancer	2.40	0.74–7.82	0.145			
Hematologic malignancy	0.63	0.08–4.85	0.658			
Respiratory failure	0.55	0.15–1.98	0.357			
End-stage renal disease	0.78	0.17–3.51	0.743			
**Chronic liver disease**	**4.91**	**1.64–1.47**	**0.004**	4.70	0.77–28.63	0.093
Severity of pneumonia						
**SOFA score**	**1.17**	**1.00–1.36**	**0.051**	1.04	0.84–1.28	0.713
**Septic shock**	**5.25**	**1.71–16.09**	**0.004**	8.33	1.57–44.27	0.013
PaO2/FiO2 ratio, mmHg	1.00	1.00–1.00	0.804			
Type of pneumonia						
VAP (vs. HAP)	2.00	0.65–6.11	0.225			
Parameters of antibiotics						
Previous antibiotic						
Class of penicillin	2.00	0.65–6.10	0.225			
Class of cephalosporin	1.29	0.35–4.67	0.703			
**Class of quinolone**	**3.55**	**1.09–1.16**	**0.035**	1.06	0.20–5.51	0.950
Class of glycopeptide	3.02	0.99–9.25	0.053			
Concomitant antibiotic						
Use of colistin	0.93	0.25–3.37	0.907			
Coverage for MRSA	1.77	0.55–5.76	0.340			
Laboratory variables						
White cell count, × 10^3/L	0.95	0.86–1.05	0.299			
C-reactive protein, mg/dL	0.97	0.90–1.05	0.499			
Procalcitonin, ng/mL	1.07	0.97–1.17	0.186			
Lactate level, mmol/L	1.09	0.97–1.22	0.133			
Organ support at baseline						
RRT	0.61	0.14–2.76	0.522			
ECMO	1.40	0.39–5.10	0.607			
Vasopressor	1.86	0.57–6.06	0.300			
Microbiologic profile						
**Bacteremia**	**8.02**	**2.60–2.47**	**<0.001**	29.48	5.36–161.99	<0.001
Pathogen of sputum culture						
**Gram(+) pathogen**	**8.65**	**1.08–6.94**	**0.042**	154.62	3.47–6885.79	0.009

Abbreviations: HR, hazard ratio; CI, confidence interval; BMI, body mass index; SOFA, sequential organ failure assessment; PaO2, partial oxygen pressure in arterial blood; FiO2, fraction of inspired oxygen; VAP, ventilator-associated pneumonia; HAP, hospital-acquired pneumonia; MRSA, methicillin-resistant *Staphylococcus aureus*; RRT, renal replacement therapy; ECMO, extracorporeal membrane oxygenation.

Table S6. Baseline characteristics of the propensity-score matched cohorts.

Variable	Extended (n = 27)	Intermittent (n = 27)	SMD
Age, y	65.48 ± 11.30	65.04 ± 13.26	0.036
Sex, female	4 (14.8)	6 (22.2)	0.192
BMI, kg/m^2	23.50 (21.47–24.97)	20.50 (18.23–23.14)	0.662
**Co-existing condition**			
Diabetes mellitus	11 (40.7)	8 (29.6)	0.234
Solid cancer	8 (29.6)	10 (37.0)	0.158
Hematologic malignancy	4 (14.8)	3 (11.1)	0.11
Congestive heart failure	3 (11.1)	1 (3.7)	0.286
Chronic liver disease	4 (14.8)	5 (18.5)	0.1
End-stage renal disease	1 (3.7)	3 (11.1)	0.286
Immunocompromised	8 (29.6)	4 (14.8)	0.362
**Severity of pneumonia**			
SOFA score	10.00 (8.00–12.50)	11.00 (9.00–13.50)	0.184
Septic shock	5 (18.5)	13 (48.1)	0.662
PaO2/FiO2 ratio, mmHg (n = 157)	179.75 (146.39–248.86)	151.00 (113.44–249.45)	0.089
**Type of nosocomial pneumonia**			
Ventilator-associated pneumonia	11 (40.7)	7 (25.9)	0.318
**Organ support at baseline**			
Renal replacement therapy	10 (37.0)	7 (25.9)	0.241
Extracorporeal membrane oxygenation	4 (14.8)	3 (11.1)	0.11
**Bacteremia**	2 (7.4)	3 (11.1)	0.128

Abbreviations: SMD; standardized mean difference, BMI, body mass index; SOFA, sequential organ failure assessment; PaO2, partial oxygen pressure in arterial blood; FiO2, fraction of inspired oxygen. Data are reported as n (%), mean ± standard deviation, or median (interquartile range).

Table S7. Baseline characteristics of patients with concurrent bacteremia according to the type of meropenem infusion.

Variable	Extended (n = 8)	Intermittent (n = 5)
Age, y	66.5 (60.0–72.3)	55.0 (35.5–71.5)
Sex, male	4 (50.0)	2 (40.0)
**Severity of pneumonia**		
SOFA score	12.0 (10.0–15.5)	11.0 (6.5–17.0)
Septic shock	1 (12.5)	3 (60.0)
**Organ support**		
Renal replacement therapy	1 (12.5)	1 (20.0)
Extracorporeal membrane oxygenation	0 (0.0)	2 (40.0)
**Type of nosocomial pneumonia**		
Ventilator-associated pneumonia	8 (100.0)	4 (80.0)
**Pathogen of bacteremia**		
Staphylococcus aureus	3 (37.5)	0 (0.0)
Enterococcus faecium	3 (37.5)	0 (0.0)
Klebsiella pneumoniae	2 (25.0)	0 (0.0)
Acinetobacter baumanii	0 (0.0)	4 (80.0)
Pseudomonas aeruginosa	0 (0.0)	1 (20.0)
**Pathogen of sputum culture**		
Staphylococcus aureus	1 (12.5)	0 (0.0)
Klebsiella pneumoniae or *oxytica*	2 (25.0)	0 (0.0)
Acinetobacter baumannii or *iwoffii*	1 (12.5)	4 (80.0)
Pseudomonas aeruginosa	0 (0.0)	1 (20.0)
**Concomitant antibiotics**		
Use of colistin	0 (0.0)	3 (60.0)
Coverage for MRSA	3 (37.5)	3 (60.0)
14-day mortality	2 (25.0)	4 (80.0)

Abbreviations: SOFA, sequential organ failure assessment; MRSA, methicillin-resistant *Staphylococcus aureus*. Data are reported as n (%), or median (interquartile range).
